# Epidemiological insights into bovine viral diarrhea virus and bovine rotavirus in diarrheic Yaks on the Qinghai-Tibet Plateau

**DOI:** 10.3389/fvets.2026.1943095

**Published:** 2026-09-29

**Authors:** Jizong Zhang, Yixin Wang, Quan Mo, Lan Lan, Yao Pan, Wei Tian, Yue Su, Yanan Zhang, Jiancheng Qi, Zhicai Zuo

**Affiliations:** 1 Key Laboratory of Animal Disease and Human Health of Sichuan, College of Veterinary Medicine, Sichuan Agricultural University, Chengdu, Sichuan, China; 2 Animal Husbandry Science Institute of Ganzi Tibetan Autonomous Prefecture, Kangding, Sichuan, China; 3 Animal Husbandry and Veterinary Station, Wa'eryi Township, Qinghai, China; 4 Agricultural and Animal Husbandry Technology Extension Service Center, Xizang, China

**Keywords:** bovine rotavirus, bovine viral diarrhea virus, diarrhea, epidemiological investigation, Yak

## Abstract

Diarrhea is common in yak calves and causes substantial economic losses to the yak industry on the Qinghai-Tibet Plateau. Bovine viral diarrhea virus (BVDV) and bovine rotavirus (BRV) are important enteric pathogens in cattle, but their epidemiology and potential association in yaks remain unclear. In this study, 1,623 fecal samples were collected from yaks across different locations, age groups, seasons, and breeds to investigate BVDV, BRV, and BVDV/BRV dual positivity. Viral positivity was detected using PCR amplification, and associated factors were analyzed using chi-square tests and multivariable binary logistic regression. Overall, 277 samples were positive for BVDV, 224 for BRV, and 92 for both viruses, corresponding to positivity rates of 17.07%, 13.80%, and 5.67%, respectively. BVDV positivity differed significantly among seasons (*P* = 0.0008), with a lower rate in summer than in spring and winter, and was strongly associated with BRV positivity (*P* < 0.0001). BRV positivity differed significantly by location, age, season, and breed in univariable analyses, and was higher in adult yaks than in calves (16.54% vs. 11.35%, *P* = 0.0025) but lower in summer than in spring and winter. Multivariable analysis showed that BRV status was the only factor associated with BVDV positivity (OR = 4.277, 95% CI: 3.121–5.861, *P* < 0.001), whereas age, sampling location, and BVDV status were associated with BRV positivity, with BVDV-positive yaks showing a markedly higher risk of BRV positivity (OR = 4.299, 95% CI: 3.138–5.889, *P* < 0.001). No factor was significantly associated with BVDV/BRV dual positivity. These findings indicate that BVDV and BRV circulate among yaks and are strongly associated, suggesting possible viral interaction or shared exposure conditions and highlighting the need for integrated surveillance and control strategies targeting multiple viral pathogens.

## Introduction

Yak (*Bos grunniens*) is a ruminant species endemic to the Qinghai-Tibet Plateau and is well adapted to high-altitude conditions, including extreme cold, hypoxia, and seasonal forage scarcity (1). Yaks provide herders with essential resources such as meat, milk, hides, fuel, and draft power, and they play an important role in sustaining pastoral livelihoods, promoting regional economic development, improving living standards, and fostering ethnic unity (2). On the Qinghai-Tibet Plateau, yak husbandry is largely based on grazing and mixed-group rearing, and their inter-herd movements across pastures, market trading, and seasonal transhumance are common, creating ecological and management conditions that facilitate the persistence and spread of respiratory and enteric pathogens (3, 4). As a result, despite the harsh environment, yaks are commonly affected by a range of infectious diseases caused by bacteria, viruses, and parasites (5, 6). These infections often compromise animal welfare, cause substantial economic losses, and hinder the sustainable development of the yak industry (7, 8). Therefore, to inform vaccine development and improve prevention and control strategies, it is essential to characterize the epidemiological profiles of major infectious pathogens in yaks.

Bovine viral diarrhea virus (BVDV) is an enveloped, positive-sense single-stranded RNA virus in the genus *Pestivirus* within the family Flaviviridea (9) that infects a wide range of even-toed ungulates, including domestic livestock such as cattle, sheep, goats, and pigs, as well as wild species such as buffalo, bison, and deer (10, 11). In cattle, BVDV infection is associated with diverse clinical outcomes, including fever, diarrhea, bloody or mucoid feces, oral and gastrointestinal mucosal lesions, reproductive failure (e.g., abortion), and other reproductive disorders, which compromise productivity and may cause mortality (12). Based on sequence variation in the 5′ untranslated region (5′-UTR), the *N*^pro^ gene, and the *E2* coding region, BVDV is classified into three genotypes with multiple sub-genotypes, including BVDV-1 (sub-genotypes a-x), BVDV-2 (sub-genotypes a-e), and BVDV-3 (also known as Hobi-like Pestivirus; sub-genotype a-e) (13, 14). Since BVDV was first described, its prevenance in China has risen from approximately 20% in the 1990s to about 57% in recent years, causing substantial economic losses (12). In addition, BVDV infection has been increasingly reported in yaks on the Qinghai-Tibet Plateau, underscoring the need for targeted prevention and control strategies (7, 8). However, available epidemiological data for BVDV in this region remain limited and, in many areas, outdated, thereby hindering vaccine development and the implementation of timely and effective prevention and control measures.

Bovine rotavirus (BRV), another major pathogen in the genus *Rotavirus* (family *Reoviridae*), possesses an 11-segments double-stranded RNA genome. This segmented genome facilitates genetic variation through point mutations and reassortment, promoting the emergence of novel strains and potentially enabling interspecies transmission (15). Based on antigenic typing of the outer capsid proteins VP4 and VP7, BRV is classified into five G genotypes (G1, G6, G8, G10, and G15) and three P genotypes (P1, P5, and P11). BRV primarily affects calves younger than 1 month, with the highest susceptibility in those under seven days of age. Infection, either alone or in combination with other pathogens, can cause acute diarrhea in young bovines, typically presenting as severe diarrhea within 12–24 h and high morbidity (90-100%), with an estimated fatality rate of approximately 50% (16). Affected calves are commonly accompanied by depression, anorexia, dehydration, and acidosis (17). In China, the predominant BRV genotypes are G6P1, G6P5, and G10P11, and the incidence of BRV-associated diarrhea in calves has been reported to be approximately 56% (18). Although BRV G6P1 has recently been reported in yaks in China (19), the epidemiological characteristics of BRV in yaks on the Qinghai-Tibet Plateau have not been systematically investigated, which is not conducive to effective prevention and control of BRV.

Hence, to investigate the epidemiological insights of BVDV and BRV in diarrheic yaks raised under a semi-grazing system on the Qinghai-Tibet Plateau, we collected watery fecal samples from yaks exhibiting clinical symptoms of diarrhea across seven counties in this region. The presence of BVDV and BRV was detected using polymerase chain reaction (PCR) analysis. This study provides evidence to support the development of effective prevention and control strategies, which may help mitigate the impact of BVDV and BRV on the yak industry on the Qinghai-Tibet Plateau.

## Materials and methods

### Experimental design

This multiregional, cross-sectional survey aimed to investigate the epidemiological characteristics of BVDV and BRV infection in diarrheic yaks raised under a semi-grazing system in seven locations on the Qinghai–Tibet Plateau between March 2024 and February 2025. The sampling sites included Nyingchi, Tibet Autonomous Region; Yushu Tibetan Autonomous Prefecture, Qinghai Province; Golog Tibetan Autonomous Prefecture, Qinghai Province; Derong County, Garzê Tibetan Autonomous Prefecture, Sichuan Province; Dege County, Garzê Tibetan Autonomous Prefecture, Sichuan Province; Daofu County, Garzê Tibetan Autonomous Prefecture, Sichuan Province; and Litang County, Garzê Tibetan Autonomous Prefecture, Sichuan Province (Figure 1). Fecal samples were collected from yaks exhibiting diarrhea-associated clinical signs, including watery or loose feces, dehydration, depression, and reduced appetite. Clinical status was assessed by local veterinarians or trained personnel at the time of sampling based on visible signs and information provided by herd owners. Only animals showing obvious diarrheic feces, with or without accompanying systemic signs, were included in this study. Relevant factors for each yak, including age, breed, sampling location, sampling season, and ambient temperature, was recorded for further analysis. Age was classified as adult (≥ 12 months) or calf (< 12 months). Breeds included Garzê–Aba–Jiulong yak (Jiulong), Tibetan alpine yak (Alpine), Qinghai Plateau yak (Plateau), and Garzê yak–cattle hybrid (Pien). Sampling locations included Nyingchi, Yushu, Golog, Derong, Dege, Daofu, and Litang. Sampling seasons were categorized as spring (March to May), summer (June to August), autumn (September to November), and winter (December to next year February). Ambient temperature data were obtained from local meteorological records corresponding to each sampling county during the sampling period.

**Figure 1:**
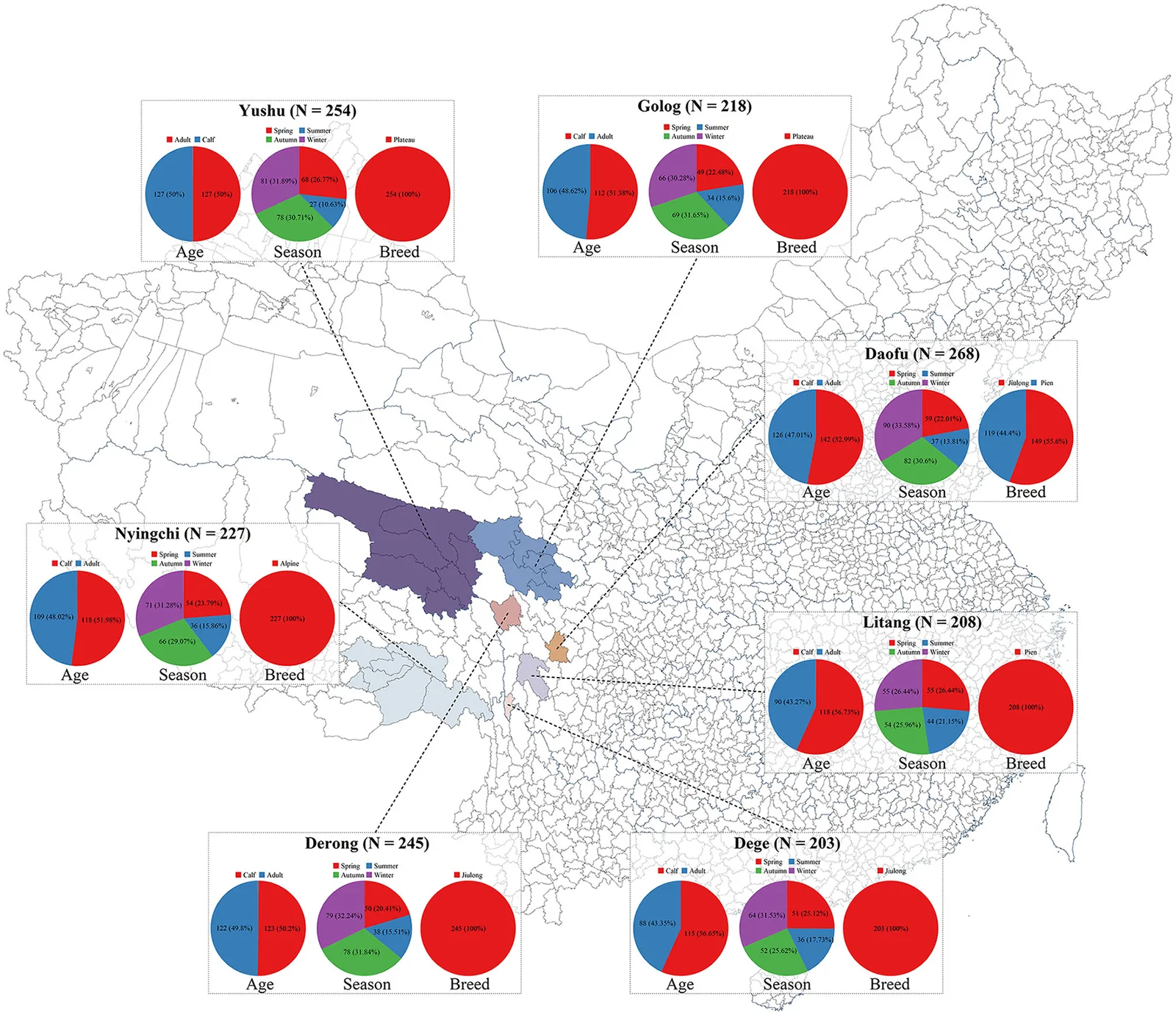
Schematic diagram showing the distribution of collected fecal samples by location, age, season, and breed.

### Sample collection and processing

Fecal samples were collected by veterinarians from local epidemic prevention departments during the study period when yaks showing obvious diarrhea-associated clinical signs were observed. Briefly, sterile anal swabs (MEDIKE GENE, Shenzhen, China) were inserted into the rectum to collect fecal specimens. All samples were placed in labeled sterile containers and transported on ice to the Sichuan Key Laboratory of Animal Disease and Human Health, Sichuan Agricultural University, for further analysis. The samples were obtained from farms/herds within the corresponding administrative regions, and all fecal samples submitted by the local veterinarians during this period were included in this study. Therefore, the sampling strategy was field-based convenience sampling targeting clinically diarrheic yaks rather than random or systematic sampling. The final sample size of 1,623 represented all eligible diarrheic yak fecal samples received by our laboratory during the study period, and no predetermined sample size was set. Upon arrival, the samples were homogenized in liquid nitrogen, diluted with physiological saline (Gibco, Beijing, China), and centrifuged at 10,000 rpm for 10 min at 4 °C. The resulting viral supernatants were collected and stored at −80 °C for subsequent analysis.

### RNA extraction and complementary DNA (cDNA) synthesis

Viral RNA was extracted from the collected viral supernatants using the MiniBEST Viral RNA/DNA Extraction Kit Ver. 5.0 (Takara Bio, Dalian, China) according to the manufacturer's instructions. The extracted viral RNA was then reverse-transcribed into cDNA using the PrimeScript^TM^ One-Step RT-PCR Kit (Takara Bio, Dalian, China) under the following conditions: 37 °C for 15 min and 85 °C for 5 s. The resulting cDNA were stored at −20 °C until further use.

### PCR amplification of the BVDV 5′-UTR and the BRV *VP6* Gene

PCR amplification was employed to detect the BVDV 5′-UTR and BRV *VP6* genes. Briefly, the resulting cDNA was used as the template for PCR amplification in a 25 μL reaction mixture containing 12.5 μL of PrimeSTAR Max Premix, 8.5 μL of RNase-free dH_2_O, 2 μL of template, 1 μL of forward primer at a final concentration of 0.4 μM, and 1 μL of reverse primer at a final concentration of 0.4 μM. PCR was performed under the following conditions: initial denaturation at 95 °C for 5 min; followed by 35 cycles of denaturation at 95 °C for 30 s, annealing at 56 °C for 30 s, and extension at 72 °C for 40 s; and a final extension at 72 °C for 10 min. The primers were designed based on published reference sequences available in the NCBI database and synthesized by Sangon Biotech (Shanghai) Co., Ltd. (Shanghai, China). Detailed information on the primers is listed in Table 1.

**Table 1:** Primers used for polymerase chain reaction amplification.

Target genes	Sequence (5'-3')	Product size (bp)	Genbank no. of reference sequence
BVDV 5'-UTR	F: CAAAATGAGAGGGGTAGCAA	248	MT179845.1
	R: CAGCAGAGATTTTTAGTAGC		
BRV *VP6*	F: TGGATGTCCTGTATTCCTTG	589	JN790188.1

The specificity and sensitivity of the primer sets were evaluated by PCR amplification. For specificity testing, cDNA extracted from BRV, BVDV, porcine epidemic diarrhea virus (PEDV), infectious bovine rhinotracheitis virus (IBRV), and Pseudorabies virus (PRV) was used as templates to determine whether the two primer sets specifically amplified their corresponding target viruses. For sensitivity testing, BRV- and BVDV-positive viral suspensions were separately subjected to 10-fold serial dilutions from 10 ^−1^ to 10 ^−6^, and cDNA extracted from each dilution was used as the template for PCR amplification. A no-template reaction mixture was included as the negative control, and cDNA extracted from BRV- and BVDV-positive viral suspensions was used as positive controls. After amplification, the PCR products were analyzed by agarose gel electrophoresis and binary outcomes (positive or negative) were obtained.

### Data processing

The positive rates of BVDV infection (BVDV^+^), BRV infection (BRV^+^), and BVDV/BRV co-infection were calculated. Co-infections status was classified as BVDV negative and BRV negative (BVDV^−^/BRV^−^), BVDV positive and BRV negative (BVDV^+^/BRV^−^), BVDV negative and BRV positive (BVDV^−^/BRV^+^), and BVDV positive and BRV positive (BVDV^+^/BRV^+^). Differences in these positivity rates across the abovementioned factors were then analyzed.

### Statistical analysis

Unless otherwise indicated, estimated proportions were reported with 95% confidence intervals (95% CIs). Data were calculated and analyzed using OriginPro 2026 software (OriginLab Corporation, Northampton, MA, USA) and SPSS 2026 software (IBM Corporation, Armonk, NY, USA). Chi-square tests were used to compare the positivity rates of BVDV infection, BRV infection, and BVDV/BRV co-infection across age groups, sampling locations, sampling seasons, and breeds. In addition, the association between BVDV positivity and BRV infection, as well as that between BRV positivity and BVDV infection, was assessed using chi-square tests. For categorical factors with more than two levels, including sampling location, sampling season, and breed, pairwise comparisons were performed when significant overall differences were detected, and the resulting *P* values were adjusted using the Bonferroni correction.

Multivariable binary logistic regression models were used to estimate odds ratios (ORs) and 95% CIs for factors including age, sampling season, sampling location, breed, and ambient temperature, and BRV or BVDV infection status where applicable. Adult yaks, autumn, Daofu, Alpine yaks, negative infection status were set as the reference categories for age, sampling location, sampling season, breed, and BRV or BVDV infection status, respectively, and ambient temperature was included as a continuous variable. Separate binary logistic regression models were constructed for BVDV infection, BRV infection, and BVDV/BRV co-infection. In the BVDV infection model, BVDV-positive samples were defined as cases and BVDV-negative samples as controls. In the BRV infection model, BRV-positive samples were defined as cases and BRV-negative samples as controls. In the co-infection model, samples positive for both BVDV and BRV were defined as cases, whereas all other samples were defined as controls. Associations between each factor and infection status were evaluated using ORs, 95% CIs, and corresponding *P* values. A *P* value < 0.05 was considered statistically significant. Data visualization was completed using OriginPro, while schematic figures were generated and edited in Adobe Illustrator and Adobe Photoshop (Adobe, San Jose, CA, USA), unless specified otherwise.

## Results

### Sample information

In this study, a total of 1,623 fecal samples were collected and examined. Among them, 227 samples (13.99%) were collected from Nyingchi, 254 (15.65%) from Yushu, 218 (13.43%) from Golog, 245 (15.10%) from Derong, 203 (12.51%) from Dege, 268 (16.51%) from Daofu, and 208 (12.82%) from Litang (Figure 1). Regarding age, 768 fecal samples (47.32%) were collected from adult yaks and 855 (52.68%) from calves. Regarding season, 386 samples (23.78%) were collected in spring, 252 (15.53%) in summer, 479 (29.51%) in autumn, and 506 (31.18%) in winter. Regarding breed, 597 samples (36.78%) were collected from Jiulong yaks, 472 (29.08%) from Plateau yaks, 227 (13.99%) from Alpine yaks, and 327 (20.15%) were Pien yaks. Detailed information on the collected samples is presented in Figure 1 and Supplementary Table 1.

### Primer specificity and sensitivity assay

The specificity and sensitivity of the primer sets were evaluated by PCR amplification. The BVDV and BRV primer sets generated specific amplification bands only from their corresponding positive template, with no amplification observed from non-target viruses or the no-template control (Supplementary Figure 1A and B). In the sensitivity assay, both primer sets produced clear amplification bands from 10^−1^ to 10^−6^ dilutions (Supplementary Figure 1C and D). Thus, the detection limits of both the BVDV and BRV primer sets were determined to be less than 10 ^−6^, confirming their suitability for detecting BVDV and BRV in clinical samples.

### BVDV positivity and risk factor analysis

Among the 1,623 fecal samples, 277 samples (277/1,623, 17.07%) were confirmed positive for BVDV by agarose gel electrophoresis analysis (Figure 2A and B). The BVDV positivity rates in samples collected from Daofu, Dege, Derong, Golog, Litang, Nyingchi, and Yushu were 17.91% (48/268), 20.20% (41/203), 12.65% (31/245), 16.51% (36/218), 17.31% (36/208), 20.70% (47/227), and 14.96% (38/254), respectively, with no significant differences detected among locations (χ^2^ = 7.8864, *P* = 0.2465; Figure 2C). The BVDV positivity rate in adult yaks (136/768, 17.71%) was comparable to that in calves (141/855; 16.49%; χ^2^ = 0.4234, *P* = 0.5152; Figure 2D). The BVDV positivity rates in spring, summer, autumn, and winter were 19.95% (77/386), 9.92% (25/252), 15.03% (72/479), and 20.36% (103/506), respectively, showing significant seasonal differences (χ^2^ = 16.6252, *P* = 0.0008). Pairwise comparison revealed that the positivity rate in summer was significantly lower than those in spring (adjusted *P* = 0.0042) and winter (adjusted *P* = 0.0018), whereas no significant differences were detected in the other comparisons (adjusted *P* ≥ 0.1734; Figure 2E). The BVDV positivity rates in Jiulong, Pein, Plateau, and Alpine yaks were 16.25% (97/597), 18.04% (59/327), 15.68% (74/472), and 20.70% (47/227), respectively, with no significant differences detected among breeds (χ^2^ = 3.2688, *P* = 0.3520; Figure 2F). The BVDV positivity rate was significantly higher in BRV-positive yaks (92/224, 41.07%) than in BRV-negative yaks (185/1,399; 13.22%; χ^2^ = 105.7884, *P* < 0.0001; Figure 2G). Because each sampling location included only breed, except for Daofu, the breed variable collapsed into two levels in the multivariable binary logistic regression analysis to avoid redundancy. The results showed that BRV infection was significantly associated with BVDV positivity (OR = 4.277; CI = 3.121–5.861; *P* < 0.001), whereas no other factors were significantly associated with BVDV positivity (*P* ≥ 0.646; Figure 2H).

**Figure 2:**
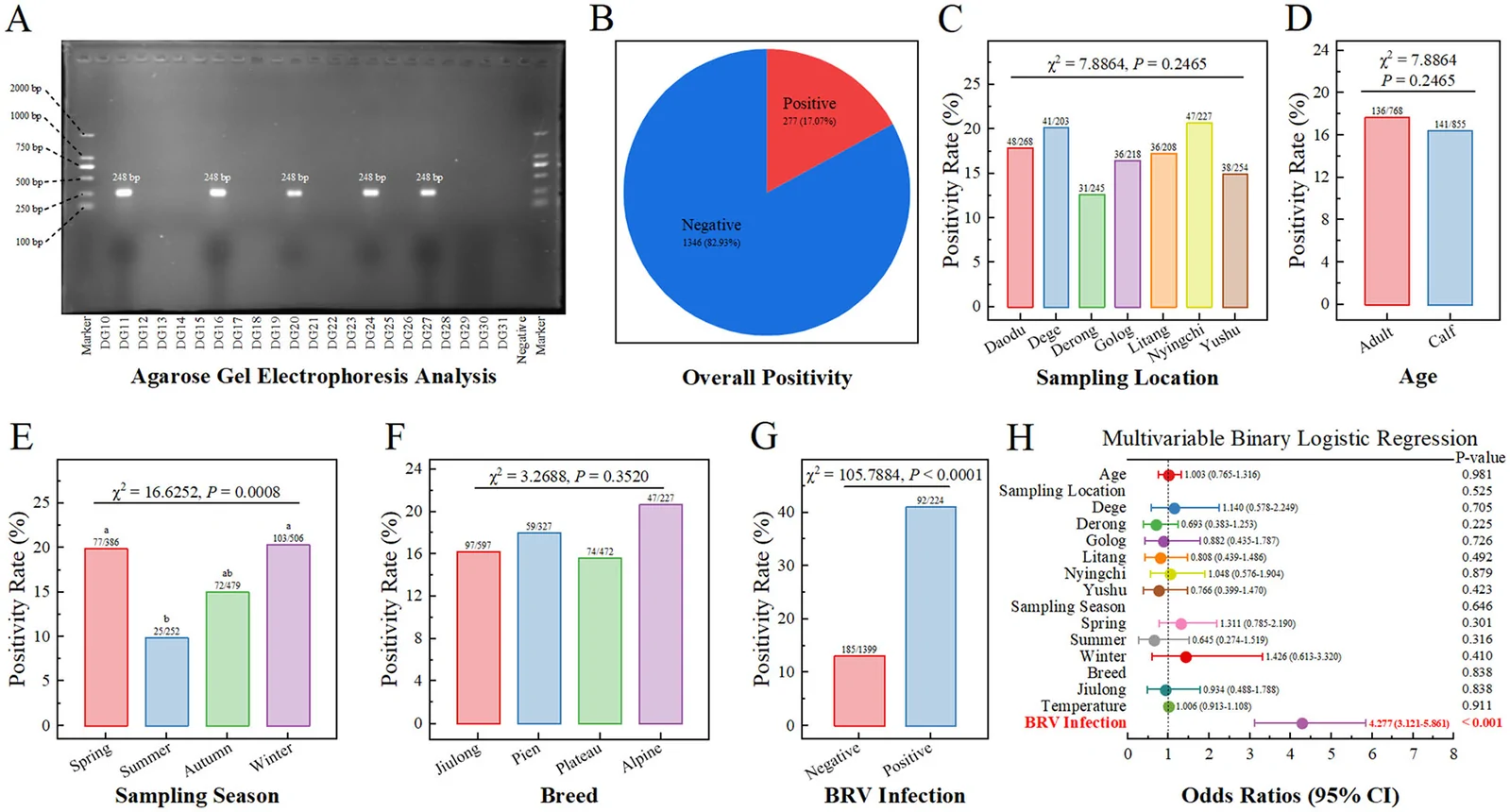
Detection and analysis of bovine viral diarrhea virus (BVDV) positivity in collected fecal samples. **(A):** Representative gel image showing the results of agarose gel electrophoresis analysis; **(B)**: Pie chart showing the number and proportion of BVDV positive samples; **(C–G)**: Bar plots showing BVDV positivity rates across different sampling locations **(C)**, age groups **(D)**, sampling seasons **(E)**, breeds **(F)**, and BRV infection **(G)**; **(H)**: Forest plot showing the associations between risk factors and BVDV positivity. In **(C–H)**, data are presented as proportions **(C–G)** or adds ratios with 95% confidence intervals **(H)**. Statistically analyses were performed using chi-square tests **(C–G)**, with pairwise comparison P values adjusted using the Bonferroni correction where applicable **(E)**, and a multivariable binary logistic regression model **(H)**. In A, lane information from left to right: Marker, DL 2000 DNA ladder; DG10-DG31, representative clinical fecal samples; Negative, negative control; Marker, DL 2000 DNA ladder. Bands at 248 bp indicate BVDV-positive samples. In **(E)**, different superscripts above the bars indicate statistically significant differences (*P* < 0.05) among groups. BRV: bovine rotavirus.

### BRV positivity and risk factor analysis

Among the 1,623 fecal samples, 224 samples (224/1,623, 13.80%) were confirmed positive for BRV by agarose gel electrophoresis analysis (Figure 3A and B). The BRV positivity rates in samples collected from Daofu, Dege, Derong, Golog, Litang, Nyingchi, and Yushu were 11.19% (30/268), 15.76% (32/203), 10.20% (25/245), 12.39% (27/218), 19.71% (41/208), 17.62% (40/227), and 11.42% (29/254), respectively. The overall chi-square test indicated a significant difference among sampling locations (χ^2^ = 15.3254, *P* = 0.0179); however, after Bonferroni correction for multiple comparisons, no pairwise comparison remained statistically significant (*P* ≥ 0.0903; Figure 3C). The BRV positivity rate was significantly higher in adult yaks (127/768, 16.54%) than in calves (97/855; 11.35%; χ^2^ = 9.1655, *P* = 0.0025; Figure 3D). The BRV positivity rates in spring, summer, autumn, and winter were 17.36% (67/386), 7.14% (18/252), 12.32% (59/479), and 15.81% (80/506), respectively, showing significant seasonal differences (χ^2^ = 16.0977, *P* = 0.0011). Pairwise comparison revealed that the positivity rate in summer was significantly lower than those in spring (adjusted *P* = 0.0012) and winter (adjusted *P* = 0.0048), whereas no significant differences were detected in the other comparisons (adjusted *P* ≥ 0.1818; Figure 3E). The BRV positivity rates in Jiulong, Pein, Plateau, and Alpine yaks were 11.73% (70/597), 17.74% (58/327), 11.86% (56/472), and 17.62% (40/227), respectively. Like the results for sampling locations, the overall chi-square test indicated a significant difference among breeds (χ^2^ = 10.6929, *P* = 0.0135); however, after Bonferroni correction for multiple comparisons, no pairwise comparison remained statistically significant (*P* ≥ 0.0684; Figure 3F). The BRV positivity rate was significantly higher in BVDV-positive yaks (92/277, 33.21%) than in BVDV-negative yaks (132/1,346; 9.81%; χ^2^ = 105.7884, *P* < 0.0001; Figure 3G). Multivariable binary logistic regression analysis identified age (OR = 0.629; CI = 0.471–0.840; *P* = 0.002), sampling location (*P* = 0.013), and BVDV infection (OR = 4.299; CI = 3.138–5.889; *P* < 0.001) as factors significantly associated with BRV positivity, whereas no other factors were significantly associated with BRV positivity (*P* ≥ 0.154; Figure 3H).

**Figure 3:**
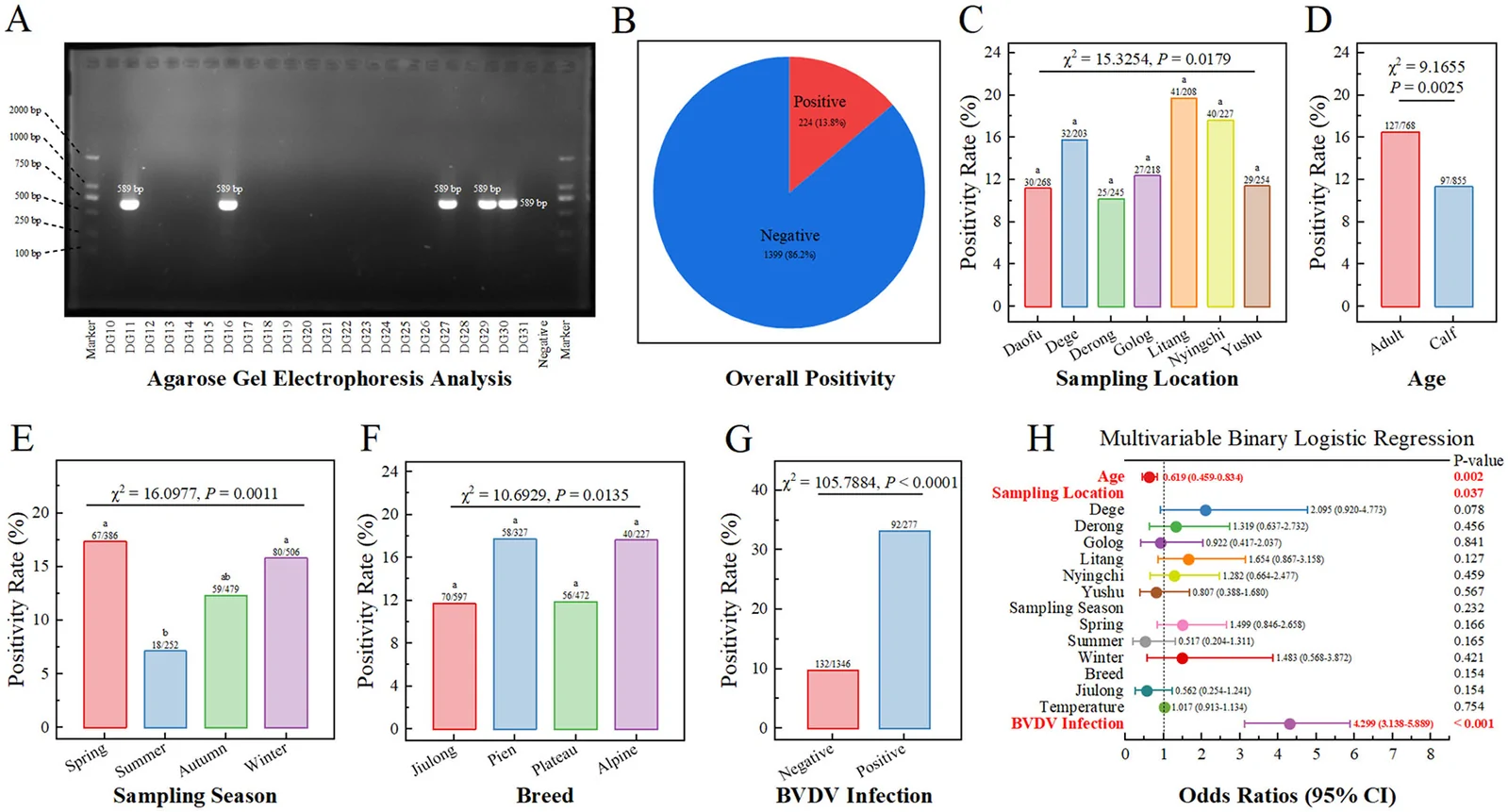
Detection and analysis of bovine rotavirus (BRV) positivity in collected fecal samples. **(A)** Representative gel image showing the results of agarose gel electrophoresis analysis; **(B)**: Pie chart showing the number and proportion of BRV positive samples; **(C–H)** Bar plots showing BRV positivity rates across different sampling locations **(C)**, age groups **(D)**, sampling seasons **(E)**, breeds **(F)**, and BVDV infection **(G)**; **(H)**: Forest plot showing the associations between risk factors and BRV positivity. In **(C–H)**, data are presented as proportions **(C–G)** or adds ratios with 95% confidence intervals **(H)**. Statistically analyses were performed using chi-square tests **(C–G)**, with pairwise comparison P values adjusted using the Bonferroni correction where applicable **(C, E, and F)**, and a multivariable binary logistic regression model **(H)**. In **(A)**, lane information from left to right: Marker, DL 2000 DNA ladder; DG10-DG31, representative clinical fecal samples; Negative, negative control; Marker, DL 2000 DNA ladder. Bands at 589 bp indicate BRV-positive samples. In **(C, E, and F)**, different superscripts above the bars indicate statistically significant differences (adjusted *P* < 0.05) among groups. BVDV: bovine viral diarrhea virus.

### BVDV/BRV daul positivity and risk factor analysis

Among the 1,623 fecal samples, 92 samples (92/1,623, 5.67%) were confirmed dual positive for BVDV and BRV by agarose gel electrophoresis analysis (Figure 4A). The dual positivity rates in samples collected from Daofu, Dege, Derong, Golog, Litang, Nyingchi, and Yushu were 7.46% (20/268), 6.40% (13/203), 5.31% (13/245), 5.05% (11/218), 5.77% (12/208), 4.41% (10/227), and 5.12% (13/254), with no significant differences detected among locations (χ^2^ = 2.8622, *P* = 0.8259; Figure 4B). The dual positivity rate in adult yaks (48/768, 6.25%) was comparable to those in calves (55/855; 5.15%) (χ^2^ = 0.9219, *P* = 0.3370; Figure 4C). The dual positivity rates in spring, summer, autumn, and winter were 5.96% (23/386), 2.38% (6/252), 5.01% (24/479), and 7.71% (39/506), respectively, showing significant seasonal differences (χ^2^ = 9.4764, *P* = 0.0236). Pairwise comparison revealed that the positivity rate in summer was significantly lower than those in winter (adjusted *P* = 0.0210), whereas no significant differences were detected in the other comparisons (adjusted *P* ≥ 0.2034; Figure 4D). The dual positivity rates in Jiulong, Pein, Plateau, and Alpine yaks were 6.03% (36/597), 6.73% (22/327), 5.08% (24/472), and 4.41% (10/227), respectively, with no significant differences detected among breeds (χ^2^ = 1.8105, *P* = 0.6127; Figure 4E). No factors were found to be significantly associated with dual positivity in the multivariable binary logistic regression analysis (*P* ≥ 0.324; Figure 4F).

**Figure 4:**
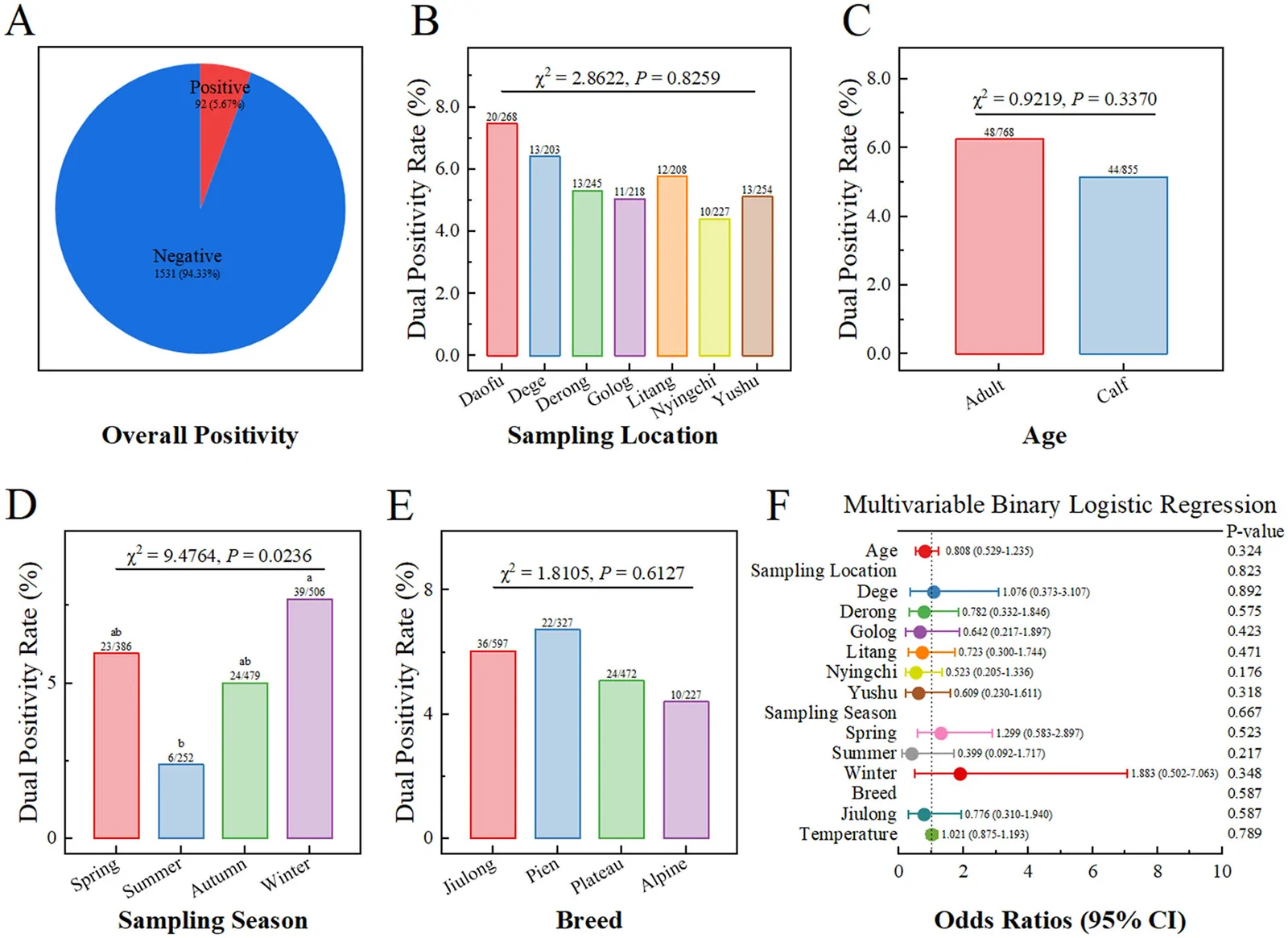
Analysis of bovine rotavirus (BRV)/bovine viral diarrhea virus (BVDV) dual positivity in collected fecal samples. **(A)**: Pie chart showing the number and proportion of BVDV/BRV dual-positive samples; **(B–E)**: Bar plots showing BVDV/BRV dual positivity rates across different sampling locations **(B)**, age groups **(C)**, sampling seasons **(D)**, and breeds **(E)**; **(F)**: Forest plot showing the associations between potential risk factors and BVDV/BRV dual positivity. In B-F, data are presented as proportions **(B–E)** or adds ratios with 95% confidence intervals **(F)**. Statistically analyses were performed using chi-square tests **(B–E)**, with pairwise comparison P values adjusted using the Bonferroni correction where applicable **(D)**, and a multivariable binary logistic regression model **(F)**. In **(D)**, different superscripts above the bars indicate statistically significant differences (adjusted *P* < 0.05) among groups.

## Discussion

Diarrhea is one of the major challenges faced by yaks, severely affecting the development of the yak industry on the Qinghai-Tibet Plateau and causing substantial economic losses. For example, the prevalence of diarrhea prevalence in adult yaks and calves has been reported to be 12.22% and 20.91% respectively, with corresponding mortality rates if 15.71% and 28.29% (20). Although multiple pathogens, including intestinal parasites, *Salmonella, Escherichia coli*, BVDV, has been identified in diarrheic yaks (20), the contributions of BVDV and BRV to disease prevalence remain unclear, hindering vaccine development and the implementation of timely and effective prevention and control measures. Therefore, in the present study, the positivity of BVDV, BRV, and BVDV/BRV dual positivity in fecal samples collected from diarrheic yaks was systematically investigated and analyzed. Notably, because the samples were collected from multiple farms/herds through field-based convenience sampling, potential herd-level clustering could not be fully excluded. Therefore, the observed positivity rates should be interpreted as detection rates among submitted diarrheic yak samples rather than population-level prevalence estimates.

### BVDV and BRV infections may contribute to the prevalence of diarrhea in Yaks

Viral pathogens, such as BVDV, BRV, bovine coronavirus (BCoV), bovine norovirus (BNoV), have been widely reported to be associated with diarrhea in domestic calves (20, 21). For example, in a case-control study investigating the microbiological etiology of calf diarrhea on cattle farms in the Midwestern United States, Cho et al. found that the positivity rate of BRV and BVDV in fecal samples from diarrheic calves were 27.1% and 0.5% (22). Recent epidemiological studies have also confirmed the circulation of BVDV and BRV in yaks (7, 19). Li et al, (23) reported that the overall antibody and antigen prevalences of BVDV in Qinghai Province from April 2024 to May 2025 were 84.52% (1,158/1,370) and 2.41% (33/1,370), respectively, indicating widespread previous exposure to BVDV and a relatively low proportion of animals with detectable antigenemia. In another study, Yan et al. (19) found that 73.6% of 541 diarrheic yak samples collected from 68 farms on the Qinghai-Tibet Plateau between April 2015 to June 2018 were positive for BRV, suggesting that BRV is an important viral pathogen associated with yak diarrhea.

In the present study, BVDV was detected in 17.07% (277/1,623) of fecal samples from diarrheic yaks (Figure 2B), which was markedly higher than the antigen prevalence reported by Li et al. (23), despite partial overlap in sampling period, sampling location, and sample size. This discrepancy may be explained by differences in the sampled populations and specimen types. First, our samples were collected specifically from diarrheic yaks, whereas Li et al. included yaks showing respiratory and/or diarrhea symptoms. Second, we detected BVDV nucleic acid in fecal samples, which may reflect intestinal viral shedding, whereas Li et al. antigen in serum, which more directly indicates systemic infection or antigenemia. Therefore, differences in clinical inclusion criteria and detection targets may have contributed to the higher BVDV positivity observed in our study. The BRV positivity rate in our study was 13.80% (224/1,623; Figure 3B), which was substantially lower than that reported by Yan et al (19). This difference may be attributable to variation in yak herds, sampling location, animal age composition, and diagnostic methods. Nevertheless, together with previous studies, our findings confirm that BVDV and BRV are widely circulating among yaks on the Qinghai-Tibet Plateau, suggesting that both viruses are involved in the pathogen spectrum of yak diarrhea and should be considered in etiological surveillance.

### Factors associated with BVDV and BRV positivity in Yaks

Viral infections in cattle are influenced by multiple host-, pathogen-, environment-, and management-related factors, including age, season, geographic region, herd density, hygiene conditions, immune status, vaccination history, and the presence of persistently infected animals (21, 24, 25). For enteric viruses, age is among the most frequently reported risk factors. BRV infection is generally more common in neonatal calves, largely because young animals have immature immune systems and are dependent heavily on adequate passive transfer of maternal antibodies (21, 26). For example, Cho and Yoon summarized that BRV, BCoV, enterotoxigenic *Escherichia coli*, and *Cryptosporidium parvum* are major pathogens associated with neonatal calf diarrhea, with BRV infection occurring most frequently during the first weeks of life (21). Similarly, Cho et al. (22) reported that BRV was significantly associated with diarrhea in calves in the Midwestern United States, with a positivity rate of 27.1% in diarrheic calves, supporting the importance of age-related susceptibility in BRV-associated diarrhea. Season is another important factor affecting viral diarrhea. BRV is generally more stable under low-temperature and humid conditions, and outbreaks of calf diarrhea are often more frequent during cold seasons, when environmental viral survival is prolonged and host resistance may be weakened (27, 28). In addition, regional differences in climate, grazing pattern, herd structure, animal movement, and biosecurity practices can further influence viral transmission (7, 25, 29). For BVDV, herd-level factors are particularly important because persistently infected animals can continuously shed virus and maintain BVDV circulation within herds.

In the present study, age, season, sampling location, and BVDV status were identified to be associated with BRV positivity in diarrheic yaks (Figure 3H). Notably, adult yaks showed a higher BRV positivity rate than calves (Figure 3D), which differs from most reports in domestic cattle, where BRV-associated diarrhea is usually concentrated in neonatal calves (20, 22). This discrepancy may be related to the unique husbandry system of yaks on the Qinghai–Tibet Plateau. Unlike intensively raised dairy or beef calves, yaks are mainly managed under extensive grazing conditions, where adult animals may have more frequent exposure to contaminated pasture, water sources, and mixed-age herds. In addition, maternal antibody dynamics in yak calves may also influence the age distribution of BRV infection, while adult yaks may experience repeated exposure, nutritional stress, pregnancy or lactation stress, long-distance grazing, and harsh climatic conditions, which could increase susceptibility to infection or promote viral shedding. Therefore, the higher BRV positivity observed in adult yaks does not necessarily indicate that adults are more clinically susceptible to BRV-associated diarrhea than calves. Rather, it may reflect differences in exposure opportunity, immune status, and subclinical viral excretion under extensive grazing conditions. However, because this study included only diarrheic animals and lacked healthy controls, and because additional matched samples could not be collected for further confirmation, the relationship between age and clinical BRV disease should be interpreted cautiously and requires further validation.

Seasonal variation was also observed in this study, with BRV positivity being lower in summer than in spring and winter (Figure 3E). This finding is consistent with the general epidemiological characteristics of enteric viruses, which can survive longer in colder environments (30, 31). In yak production areas, winter and spring are often associated with low temperature, limited forage availability, poor nutritional status, and increased physiological stress, all of which may reduce host resistance and facilitate viral transmission. In contrast, higher temperatures, stronger ultraviolet radiation, and improved forage availability in summer may reduce environmental viral persistence and enhance host condition. Similar seasonal effects have also been described for BVDV infection in yaks. Li et al. (23) reported that BVDV occurrence in yaks in northwest China showed seasonal characteristics, with infections occurring frequently during periods of intensive grazing and animal movement. Although their study focused on BVDV rather than BRV, it supports the idea that seasonal grazing behavior and environmental stress can influence viral infection dynamics in yak populations. Notably, although ambient temperature is related to season, it was retained in the analysis because it may reflect additional environmental variation associated with geographic factors such as latitude, longitude, altitude, and local microclimate. Unlike season, which represents broad temporal categories, temperature is a continuous variable and may capture location-specific or short-term climatic effects on viral persistence, host stress, and transmission risk. Nevertheless, because temperature and season may be correlated, their effects should be interpreted cautiously.

Sampling location was another factor associated with BRV positivity in our study (Figure 3C). This result may reflect regional differences in altitude, temperature, precipitation, herd density, grazing routes, water sources, management practices, and local disease prevention measures. Previous studies have shown that viral infections in yaks vary substantially among regions. For example, Diao et al. (7) conducted a systematic review and meta-analysis of BVDV infection in yaks in mainland China and found obvious regional variation in BVDV prevalence, suggesting that geographic and management-related factors are important determinants of viral circulation in yak populations. Similarly, Yan et al. (19) reported a high BRV positivity rate in diarrheic yak samples collected from multiple farms on the Qinghai–Tibet Plateau, indicating that BRV infection is widespread but may differ among herds and areas. In our study, breed and sampling location may have been partly confounded because certain yak breeds are distributed in specific geographic areas. Therefore, apparent differences among breeds or locations should be interpreted carefully, as they may reflect environmental exposure and management conditions rather than intrinsic host susceptibility alone.

For BVDV positivity, BRV infection was the only significant factor retained in the multivariable model, whereas age, season, sampling location, breed, and temperature were not independently associated with BVDV positivity (Figure 2H). This result suggests that BVDV infection in diarrheic yaks may be less dependent on individual demographic factors and more strongly influenced by herd-level transmission dynamics. This interpretation is consistent with the known epidemiology of BVDV, in which persistently infected animals, animal introduction, herd contact patterns, vaccination status, and biosecurity level are major drivers of viral maintenance (23). The absence of significant associations with conventional risk factors in our study may also be related to limitations in available epidemiological information. For example, we did not determine whether BVDV-positive animals were persistently or transiently infected, nor did we collect detailed data on vaccination history, herd size, animal movement, colostrum intake, nutritional condition, or the presence of other enteric pathogens. These unmeasured factors may have affected BVDV detection and could partly explain why only BRV status remained significant in the final model.

Overall, the risk-factor analysis suggests that BRV infection in diarrheic yaks is influenced by host age, season, geographic location, and concurrent BVDV infection, whereas BVDV positivity appears to be more closely associated with BRV status than with the measured demographic or environmental variables. Compared with previous studies in domestic calves, the risk pattern observed in yaks may reflect the distinct ecological environment and extensive grazing management of the Qinghai–Tibet Plateau. These findings indicate that prevention and surveillance of yak viral diarrhea should not focus only on calves, but should also consider adult animals, seasonal transmission peaks, regional differences, and potential viral co-infections.

### Potential synergistic interaction between BVDV and BRV

The significant association between BVDV and BRV positivity observed in this study suggests a potential interaction between these two viruses in diarrheic yaks. In the multivariable analysis, BVDV status was associated with BRV positivity (Figure 3H), whereas BRV status was the only factor retained in the final model for BVDV positivity (Figure 2H), indicating that co-infection may occur more frequently than expected by chance. Although the cross-sectional design precludes causal inference, this association is biologically plausible. BVDV is well recognize for its immunosuppressive effects, which involve impairment of both innate and adaptive immune responses and may increase host susceptibility to secondary infection (32, 33). Persistently infected animals are an important reservoir of BVDV because they can shed virus continuous and sustain viral circulation within herds (34, 35). By contrast, BRV primarily infects mature enterocytes on the small intestine villi, causing epithelial damage, villous atrophy, malabsorption, and diarrhea (36, 37). Therefore, BVDV-induced immunosuppression may facilitate BRV infection, while BRV-induced intestinal damage may further compromise mucosal integrity and aggravate diarrhea in co-infected animals.

This potential interaction may be particularly important in yak production systems on the Qinghai–Tibet Plateau. Yaks are commonly raised under extensive grazing conditions, where mixed-age herds share pastures and water sources, increasing environmental contamination and repeated exposure to enteric viruses. Seasonal nutritional stress, cold temperatures, pregnancy- or lactation-related stress, long-distance grazing, and limited biosecurity may further reduce host resistance and promote viral transmission. Thus, the observed association between BVDV and BRV may reflect both biological interactions and shared epidemiological factors. However, because this study included only diarrheic animals and lacked healthy controls, viral load data, PI animal identification, and screening for other enteric pathogens, the clinical significance of BVDV/BRV co-infection should be interpreted cautiously. Future studies incorporating these data are needed to clarify the role of co-infection in yak diarrhea.

## Conclusion

In conclusion, this study demonstrated that both BVDV and BRV are detectable in fecal samples from yaks and that their positivity statuses are strongly associated. BRV positivity was associated with age, season, sampling location, and BVDV infection status, whereas BVDV positivity was mainly associated with BRV status. Although no independent risk factor was identified for BVDV/BRV dual positivity, the detection of dual-positive samples indicates that mixed viral infection should not be overlooked in yak populations. These findings provide epidemiological evidence for the circulation and potential interaction of BVDV and BRV in yaks and highlight the importance of integrated surveillance and control strategies targeting multiple enteric pathogens rather than individual viral agents alone.

## Data Availability

The original contributions presented in the study are included in the article/Supplementary material, further inquiries can be directed to the corresponding authors.
